# Resonance Frequency of Optical Microbubble Resonators: Direct Measurements and Mitigation of Fluctuations

**DOI:** 10.3390/s16091405

**Published:** 2016-08-31

**Authors:** Alessandro Cosci, Simone Berneschi, Ambra Giannetti, Daniele Farnesi, Franco Cosi, Francesco Baldini, Gualtiero Nunzi Conti, Silvia Soria, Andrea Barucci, Giancarlo Righini, Stefano Pelli

**Affiliations:** 1 Museo Storico della Fisica e Centro Studi e Ricerche Enrico Fermi, Piazza del Viminale 1, Rome 00184, Italy; d.farnesi@ifac.cnr.it (D.F.); gnc@ifac.cnr.it (G.N.C.); g.c.righini@ifac.cnr.it (G.R.); s.pelli@ifac.cnr.it (S.P.); 2 IFAC-CNR, Istituto di Fisica Applicata “Nello Carrara”, Consiglio Nazionale delle Ricerche, Via Madonna del Piano 10, Sesto Fiorentino 50019, Italy; s.berneschi@ifac.cnr.it (S.B.); a.giannetti@ifac.cnr.it (A.G.); f.cosi@ifac.cnr.it (F.C.); f.baldini@ifac.cnr.it (F.B.); s.soria@ifac.cnr.it (S.S.); a.barucci@ifac.cnr.it (A.B.)

**Keywords:** microresonators, whispering gallery modes, stability, fluctuations, stabilization

## Abstract

This work shows the improvements in the sensing capabilities and precision of an Optical Microbubble Resonator due to the introduction of an encaging poly(methyl methacrylate) (PMMA) box. A frequency fluctuation parameter σ was defined as a score of resonance stability and was evaluated in the presence and absence of the encaging system and in the case of air- or water-filling of the cavity. Furthermore, the noise interference introduced by the peristaltic and the syringe pumping system was studied. The measurements showed a reduction of σ in the presence of the encaging PMMA box and when the syringe pump was used as flowing system.

## 1. Introduction

Optical Microbubble Resonators (OMBRs) are a novel type of microresonator with embedded microfluidic [1,2]. Due to their high Q-Factor, over 1 × 10^7^ [2], OMBRs are suitable for sensing applications of biomarkers [3,4], chemicals [5], temperature [6], and pressure [7,8]. An OMBR consists of a spherical silica shell created by an arc voltage discharge applied to an inflated silica capillary. Melted silica isotropically expands under the effect of an internal slight overpressure, around 0.2 bar, producing a bubble, which can act as a resonator. Light coupled inside the OMBR becomes trapped by total internal reflection. For sensing applications, the most used coupling system is represented by the evanescent field coupled out of a tapered fiber [1,2,3,4,5,6,7]. Exactly as in massive microspherical resonators, the so-called Whispering Gallery Modes (WGM) build up inside the cavity. Low losses and high power concentration can give rise to non-linear phenomena such as the Kerr effect [9] and third harmonic generation [10]. Sensing sensitivity and efficiency are directly related to resonator Q-Factor and resonance stability. Recently, new detection limits have been achieved by measuring the line shift and the broadening of the resonance simultaneously [11,12]. Indeed, resonance thermo-mechanical fluctuations could interfere with the experiment. For example, a thermal resonance drift could hide or overestimate the shift due to the presence of a target molecule or chemical component. In recent years, different solutions have been proposed for stabilizing both mechanical coupling and temperature. One approach is based on a transparent box that encloses the resonator and the tapered fiber [13,14]. In another one, the resonator and the fiber are bonded by means of an optical glue that ensures, besides a thermal insulation, a well proved mechanical stability [15,16,17]. In any case, all the articles mentioned above were mainly concentrated on the Q-Factor of the packaged system or on its temperature sensing capabilities under external mechanical noise [14], without making a proper analysis of resonance fluctuations before and after the packaging [8]. In this work, we propose a PMMA surrounding box with two grooves for orthogonal coupling between the OMBR and the tapered fiber. We focused our investigation on the frequency fluctuations of the OMBR resonance versus the elapsed time. We started comparing the amplitudes of the fluctuations with or without the box and with or without the flowing fluid inside the resonator. A fast and automatized data acquisition procedure of resonance spectra through a data acquisition (DAQ) board that also allowed constant monitoring of the room temperature was developed. The study of the resonator with a fluid inside is justified by the peculiar application of OMBRs as optical platforms for chemical and biochemical sensors, where the fluid is the reference medium before and after the reaction takes place into the hollow cavity. With this perspective, we closely analyzed the interference produced by two different fluid pumping systems, namely peristaltic and syringe pumps.

## 2. Materials and Methods

### 2.1. Silica Glass OMBR

Silica glass capillaries were placed at the center of four electrodes with a squared arrangement. Capillaries were inflated with a pressure of 1.2 bar. Two sequential arc discharges between the electrodes caused the silica melting, followed by isotropic expansion giving rise to OMBR. Three microbubbles were used in this experiment, with thicknesses ranging from 5 µm to 7 µm [18]. The measured Q-Factors were in the order of 1 × 10^7^ in the case of air-filling, and in the order of 1 × 10^6^ in the case of water-filling. Q-Factor was measured at a wavelength of 1500 nm.

### 2.2. Coupling and Enclosing System

**Figure 1:**
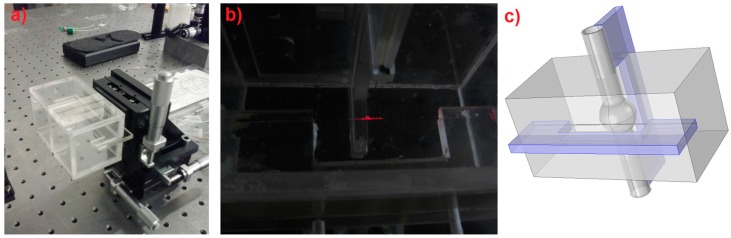
(**a**) Photo of the PMMA box that encloses the OMBR and the tapered fiber around the coupling area. Two orthogonal grooves allow the fine movements of the two C supports that are responsible to hold the resonator and the fiber. (**b**) Photo of the coupling area in which the taper and the OMBR are clearly visible due to scattered red light out of a diode laser. (**c**) 3D sketch of the box: two orthogonal holders (in violet) are used to allow the coupling between the OMBR and the tapered fiber.

Light coupling into the resonator was obtained by evanescent wave from a tapered fiber. The OMBR and the fiber were each mounted on its own C-shaped support, which were orthogonal to each other. The supports could be finely moved in micrometric stages. Coupling took place when the resonator and the fiber were in close contact. Fine adjustment of the reciprocal position enabled us to find the best coupling condition. A specially designed PMMA box was used to encage the coupling area, as shown in Figure 1a. The photo in Figure 1b refers to the center of the box where the coupling occurs. Two orthogonal grooves allowed for the insertion of the supports inside the box, as depicted in Figure 1c.

In the case of the water-filling of the resonator, it was reasonable to assume that the resonance used in this work were mainly confined inside the silica shell of the resonator. Indeed, in all cases, a red shift was observed while the temperature increased, which was in accordance with a mode mainly confined inside the fused silica, showing a positive value of the thermo-optic coefficient, contrarily to the negative value of water [19].

### 2.3. Lasers and Detectors

In this experiment, we employed a 1500-nm ECL diode laser source (Tunics Reference, Anritsu, Japan) directly coupled to the tapered fiber. An external voltage modulation allowed us to change the frequency output from the laser (300 MHz/V). Light coupling and resonance spectra were observed by detecting the transmission of the tapered fiber. For the signal detecting, a nanosecond IR photodetector (1623, New Focus, Irvine, CA, USA) was used.

### 2.4. Acquisition System

Frequency modulation of the laser light occurred by applying an external triangular voltage wave of 10 Vpp, corresponding to a modulation of about 3 GHz. The triangular wave was generated by means of a National Instruments DAQ board (USB-6211, National Instruments, Austin, TX, USA). The DAQ board was also used for acquiring the transmitted power through the tapered fiber. The entire synchronization was managed by a LabView program (LabView, National Instruments, Austin, TX, USA). A triangular wave was sent to the laser every second, and the correspondent transmission was recorded on a laptop. Room temperature was monitored with a LM35 (Texas Instruments, Dallas, TX, USA, sensitivity 10 mV/°C), directly connected to the DAQ board. In the case of measurements involving the PMMA box, an additional temperature sensor, placed inside the box, was used.

### 2.5. Pumping Systems

Two different pumping systems, connected to the microbubble capillary through a Tygon^®^ tube, were used for the experiments. One was a peristaltic pump (Minipuls 3, Gilson), operating in a tunable range of 1 to 5 rounds per second that corresponded to fluid flowing speed in the range 10–50 µL/min. The second system made use of a syringe pump, with the flow rate ranging from 20 up to 60 µL/min.

### 2.6. Data Analysis

All the acquired spectra were analyzed as a function of the time through a Matlab program (Mathworks, Massachusetts, MA, USA). For each spectrum, a single resonance was selected. The resonance frequency was easily obtained by converting the corresponding voltage applied to the laser into the resonance position. The resonance position was automatically identified by the program, and all resonance frequencies were recorded as a function of the elapsed time. For a better interpretation of the graph reported, the reader can refer to Videos V1 and V2 in the Supplementary Materials. In order to obtain a score of frequency fluctuation, we introduced the parameter σ, which represents the standard deviation of the resonance fluctuations in a time window of 20 s, as described in the following equation:

(1) $$\sigma =\sqrt{\overset{20}{\underset{t=\;1}{\sum }}\frac{{\left(f_{t}-\bar{f}\right)}^{2}}{20}\;,}$$

where *f_t_* is the measured frequency of the resonance peak at time *t*, and $\bar{f}$ is the average of the frequency in the 20-s time window. The value reported for each measurement is the average of the entire acquisition time.

To have a homogeneous representation of the measured values, all graphs reported refer to a single OMBR. Measurements on the other resonators showed similar qualitative behavior.

## 3. Results and Discussion

### 3.1. Static Resonance Stability of the OMBR System Coupled in Air

**Figure 2:**
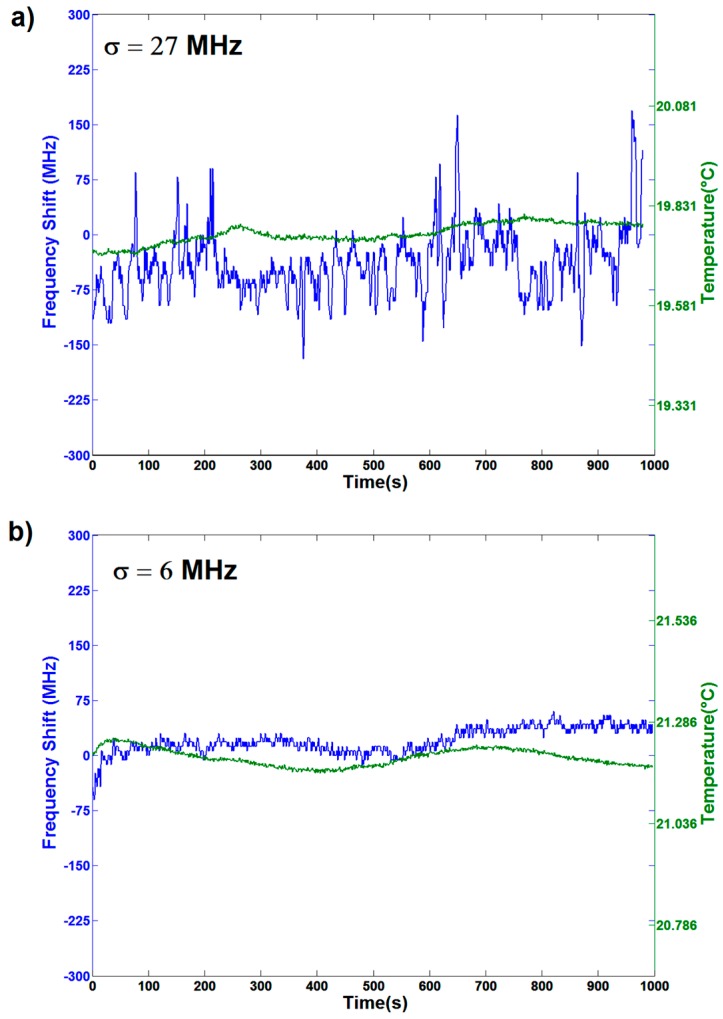
Resonance frequency (blue curves) and temperature fluctuations (green curves) versus elapsed time for an open space coupling in the case of air- (**a**) and water- (**b**) filling of the resonator. The correspondent value of σ is 27 MHz in the former case (**a**), and it reduces to 6 MHz in the latter (**b**). In the case of water-filling, a direct correlation of resonance frequency and temperature oscillation is clearly observable.

As a first step, the resonance frequency fluctuations of the coupled OMBR in air were analyzed without the packaging box. Oscillation was mainly caused by mechanical [13,14] and thermic [20,21,22,23,24,25] effects. In the case of air-filled capillary (see Figure 2a), the frequency fluctuations presented a variance σ = 27 MHz. The presence of water in the OMBR increased both its mechanical and thermal inertia. As a final result, the fluctuation was damped down, and the variance was reduced to σ = 6 MHz (see Figure 2b). This latter case represents the real working condition of OMBR sensors of biomarkers and chemical species. The temperature reported was acquired next to the coupling system (in green). It can be observed that the room temperature showed an oscillation of about 0.1 °C. These oscillations are due to the laboratory air conditioning system that introduces a hysteresis in order to keep the temperature constant.

It can also be observed that the resonance frequency oscillated with the same frequency of the temperature, but with a time-phase delay due to the thermal properties of the OMBR and the water-filling. The amplitude of these oscillations was in the order of 30–40 MHz.

### 3.2. Static Resonance Stability When OMBR System is Encased

**Figure 3:**
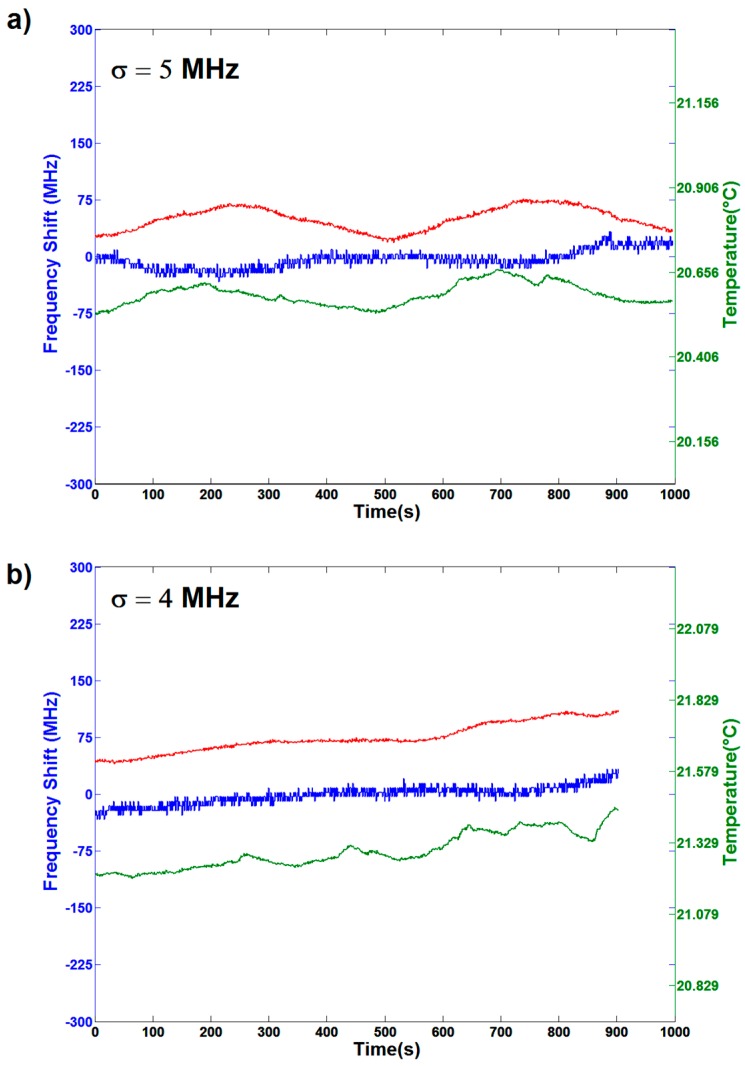
Resonance frequency and temperature fluctuations versus elapsed time for the OMBR system enclosed in a PMMA box in the case of air-(**a**) and water-(**b**) filling of the resonator. The corresponding values of σ for similar temperature oscillations are 5 MHz and 4 MHz in the case of air-filling and water-filling, respectively. A direct correlation of resonance frequency and temperature oscillation is clearly observable. Such oscillations are damped in the case of water-filling.

When the whole coupling system is enclosed in a PMMA box, a strong diminution of the resonance fluctuations can be observed (Figure 3). The measured σ values are 5 MHz and 4 MHz in the cases of air-filling and water-filling, respectively. The temperature was measured in two different places, inside the box (red curve) and outside, in its proximity (green curve): as expected, the former is smoother than the latter. In the case of the air-filled OMBR (Figure 3a), we can still see that the resonance drift follows the room temperature oscillation, while the water-filled resonator (Figure 3b) is less influenced by fast oscillation, slowly following the thermal drift. In the case of the air-filling in the absence of the box, oscillations were well above the resonance bandwidth (20 MHz), leading to a reduction of the effective sensitivity. The noticeable reduction of the resonance fluctuations when the system is encased leads us to conclude that the PMMA box mainly damps the mechanical vibrations due to the airflow that makes the coupling distance between the OMBR and the tapered fiber unstable. A further reduction of the σ value also in the water-filling case leads us to deduce that the thermal oscillations are also damped by the presence of the enclosing system.

The difference of the temperature values in the two measurements is mostly due to the accuracy of the sensor used. Even if it is not possible to give an absolute value of the temperature, the variations measured with the two systems are consistent with each other.

### 3.3. Dynamic Resonance Stability When Using a Peristaltic Pump

**Figure 4:**
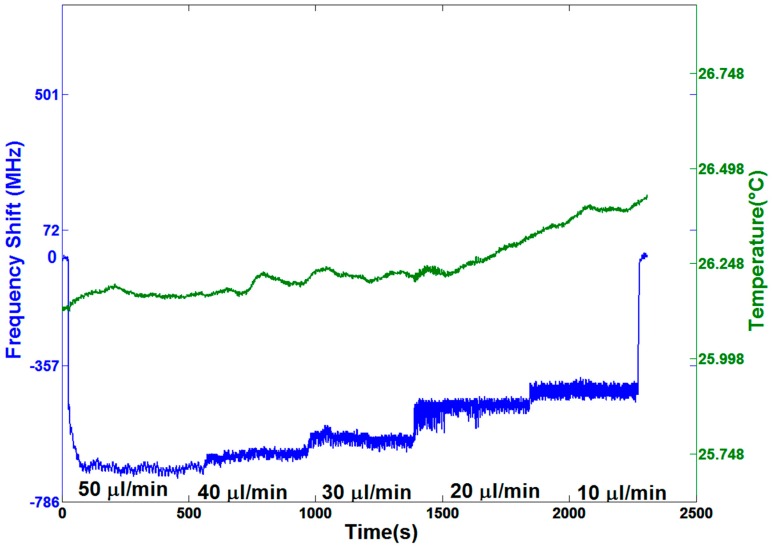
Resonance frequency shifts and temperature fluctuations versus elapsed time, with water flowing at different pumping speeds, starting with no flow and ranging from 50 µL/min down to 10 µL/min, produced by a peristaltic pump. There is a different frequency shift for each pumping speed. Furthermore, the fluctuation parameter σ decreases with the increase of the speed, which might originate from the low pass frequency behavior of the Tygon tubing.

With the purpose of analyzing the stability of the sensor while the analyte fluid is flowing inside the hollow resonator cavity, the effect of the pumping system on the resonance stability was studied. First, we analyzed the influence of a peristaltic pump working at different velocities. The pumping speed varied from 5 round/min (50 µL/min) down to 1 round/min (10 µL/min). Video V1 in the Supplementary Materials shows the graph creation by following the resonance position. It can be observed that the water flow changes the internal pressure inside the resonator and therefore causes a resonance shift [7,8]. Indeed, a clearly different resonance shift for each pumping speed is noticeable (Figure 4). Moreover, fast oscillations are also observable. They were introduced by the pump, since its working principle involves the water hammer effect, in which the overall pressure has an average effect on pressure burst. Higher harmonics frequencies are thus produced by the compression and resilience stages of the Tygon tube. It is well-known that the flow induced by peristaltic pumps is pulsed, particularly at low rotational speeds. As a matter of fact, for higher pumping speeds, the higher frequency components are reduced and the variance σ is equal to 8.2, whereas, for a slow flow rate such as 10 µL/min, the resonance shows higher fluctuations (σ = 19).

It can be observed that the frequency shifts do not increase linearly with the flowing speed. This is due to the pressure drop originated from the fluid velocity inside the microfludic tube. This contribution can be divided into two components: The first one is related by the simple Bernoulli equation, and the second one is related to the friction that the water encounters inside the microfluidic channel. Indeed, the capillary used shows a high ratio between the fused silica roughness and the tube diameter [26]. Both components show a dependence on the square velocity, explaining the lack of a linear response.

The frequency shift due to the pumping pressure should be carefully taken into account while performing biochemical sensing applications. Indeed, it might be necessary to change the solution inside the resonator and therefore switch the pumping system off and on, causing a resonance shift that can interfere with the measurements.

### 3.4. Dynamic Resonance Stability When Using a Syringe Pump

**Figure 5:**
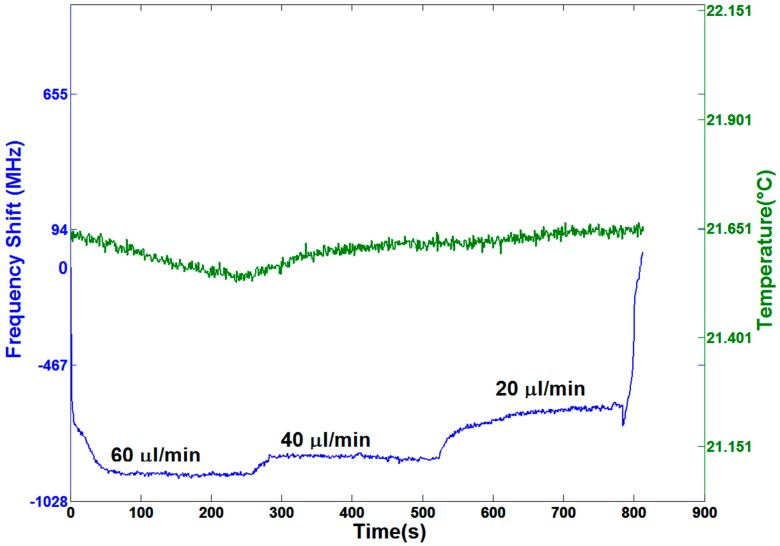
Resonance frequency shifts and temperature fluctuations versus elapsed time with water flowing at different pumping speeds starting with no flow and ranging from 60 µL/min up to 20 µL/min with steps of 20 µL/min, as produced by a syringe pump. A different shift for each pumping speed is clearly observable. The correspondent fluctuation parameter σ is the same as the one with the pump switched off. In this case, the variations induced by thermal fluctuations are negligible.

Thanks to the different working principle, a syringe pump, in which the pumping pressure is created by a steady movement of the syringe piston, allows a constant water flow and therefore does not induce high-frequency oscillations. In Figure 5, the pumping speed is varied from 60 µL/min down to 20 µL/min in steps of 20 µL/min. Graph creation though resonance position is shown in Video V2 in the Supplementary Materials.

It is clearly observable that frequency fluctuation (σ = 4.2 MHz) is reduced compared with the case of the peristaltic pump. This value is comparable with the one obtained in the static case (pump switched off), so we can conclude that this pumping method does not affect the accuracy of sensing measurements. Finally, it can be pointed out that the system requires around 100 s in order to reach a pressure stability; this might be explained by the delayed deformation of the Tygon tubing due to its intrinsic elasticity. The peak observable at the end of the period with a pump rate of 20 µL/min could be due to abruptly switching off the pump.

This work examined the improvements in the stability of a tapered fiber-OMBR coupling system for sensing applications achievable by the use of a PMMA transparent box that encloses the coupling volume. In a previous study, Dong et al. tested the performance of a microcylinder resonator for temperature sensing under induced mechanical noise [14]. They demonstrated that the presence of an encasing box isolated the coupling system from external mechanical vibrations and therefore improved the sensing capabilities. However, most of the studies had focused on sensing performances, without considering the fluctuations in detail [14,15,16]. Furthermore, the final resolution is often evaluated by the precision that could be obtained by a WGM with a certain Q-Factor based on the slope of a calibrating curve. Usually the precision of the frequency shift measurement comes out as 1/100–1/50 of the resonance bandwidth (strictly related to the Q-Factor) [2,27]. Final precision is then evaluated by multiplying this factor by the calibrating curve slope. This approach does not take into account possible fluctuations of the resonance that could interfere with the precision of the measurements. A study concentrated on the analysis of resonance fluctuations, as an important parameter for microresonator-based sensors, was proposed by Ma et al. [24], but merely on a qualitative approach. In this work, we deepened the analysis, with specific reference to OMBRs, and introduced a short time–frequency fluctuation variance σ as the score parameter for coupling steadiness. A similar approach on a long time scale of 10 min was used by Madugani et al. in a study on a pressure sensor involving an OMBR [8]. We demonstrated that significant improvements in the sensing stability are achieved when using a PMMA box to protect the coupling volume, leading to a reduction of σ by a factor of 2. Furthermore, we observed that, in the case of an OMBR, liquid-filling of the hollow cavity makes the sensor less sensitive to room temperature and mechanical oscillations. Bearing in mind chemical- and biomarker-sensing applications, we studied the noise related to two different pumping systems: A peristaltic pump introduces high harmonics noise that negatively affects the variance σ; the value of σ in this case is comparable to the one obtained for the system in air. On the contrary, a syringe pump, even if it requires a certain stabilization time, does not introduce any additional noise and therefore is more suitable for chemical- and biomarker-sensing applications.

## 4. Conclusions

In this work, we analyzed and compared the resonance frequency fluctuations, in the presence and absence of a PMMA box that encloses the coupling volume of a sensor based on an optical microbubble resonator (OMBR). These oscillations in turn strongly determine the sensor capabilities. The introduction of the box notably reduced the total fluctuations, as demonstrated by the decrease of the frequency fluctuation factor σ. Similar behavior of the fluctuations were also found in the other characterized OMBRs.

In the case of the air-filled resonator, the observed resonance frequency fluctuations are considerably damped down by the presence of water inside the microbubble: this could arise from a higher thermal and mechanical inertia. This evidence is an important advantage of OMBR-based sensors, where the fluid containing the analyte flows inside the capillary and the microbubble. In any case, the frequency oscillation due to temperature hysteresis was found to be in the order of 30–40 MHz. Even if these fluctuations do not strongly affect measurements on macroscopic effects as refractive index or temperature, where measured shifts are in the order of hundreds of MHz, they may play an important role in the case of the detection of very low chemical and biochemical concentrations. In this latter case, a system for temperature control and stabilization must be introduced in the set-up to guarantee the required sensitivity.

Finally, two different pumping methods were also tested: while a peristaltic pump introduces high harmonic oscillations that may interfere with the sensing experiment, a syringe pump guarantees a continuous flow, producing an oscillation variance comparable to the static case and is therefore more practical for use with sensors.

## Supplementary Materials

The following are available online at http://www.mdpi.com/1424-8220/16/9/1405/s1. Video V1: Resonance Frequency shift Vs peristaltic pump speed; Video V2: Resonance Frequency shift Vs syringe pump speed.

## References

[1] Sumetsky M., Windeler R.S., Dulashko Y. (2010). Optical microbubble resonator. Opt. Lett..

[2] Berneschi S., Farnesi D., Cosi F., Conti G.N., Pelli S., Righini G.C., Soria S. (2011). High Q silica microbubble resonators fabricated by arc discharge. Opt. Lett..

[3] Zhang X., Liu L., Xu L. (2014). Ultralow sensing limit in optofluidic micro-bottle resonator biosensor by self-referenced differential-mode detection scheme. Appl. Phys. Lett..

[4] Li H., Fan X. (2010). Characterization of sensing capability of optofluidic ring resonator biosensors. Appl. Phys. Lett..

[5] Stoian R.I., Bui K.V., Rosenberger A.T. (2015). Silica hollow bottle resonators for use as whispering gallery mode based chemical sensors. J. Opt..

[6] Ward J.M., Yang Y., Chormaic S.N. (2013). Highly Sensitive Temperature Measurements with Liquid-Core Microbubble Resonators. IEEE Photon. Technol. Lett..

[7] Henze R., Seifert T., Ward J., Benson O. (2011). Tuning whispering gallery modes using internal aerostatic pressure. Opt. Lett..

[8] Madugani R., Yang Y., Le V.H., Ward J.M., Chormaic S.N. (2016). Linear Laser Tuning Using a Pressure-Sensitive Microbubble Resonator. IEEE Photon. Technol. Lett..

[9] Li M., Wu X., Liu L., Xu L. (2013). Kerr parametric oscillations and frequency comb generation from dispersion compensated silica micro-bubble resonators. Opt. Express.

[10] Farnesi D., Barucci A., Righini G.C., Berneschi S., Soria S., Nunzi G. (2014). Optical Frequency Conversion in Silica-Whispering-Gallery-Mode Microspherical Resonators. Phys. Rev. Lett..

[11] Shen B.Q., Yu X.-C., Zhi Y., Wang L., Kim D., Gong Q., Xiao Y.-F. (2016). Detection of Single Nanoparticles Using the Dissipative Interaction in a High-Q Microcavity. Phys. Rev. Appl..

[12] Shao B., Jiang X.-F., Yu X.-C., Li B.-B., Clements W.R., Vollmer F., Wang W., Xiao Y.-F., Gong Q. (2013). Detection of Single Nanoparticles and Lentiviruses Using Microcavity Resonance Broadening. Adv. Mater..

[13] Dong Y., Wang K., Jin X. (2015). Packaged microsphere-taper coupling system with a high Q factor. Appl. Opt..

[14] Dong Y., Jin X., Wang K. (2015). Packaged and robust microcavity device based on a microcylinder-taper coupling system. Appl. Opt..

[15] Wang P., Ding M., Murugan G.S., Bo L., Guan C., Semenova Y., Wu Q., Farrell G., Brambilla G. (2015). Packaged, high-Q, microsphere-resonator based add-drop filter. Opt. Lett..

[16] Yan Y.-Z., Zou C.-L., Yan S.-B., Sun F.-W., Ji Z., Liu J., Zhang Y.-G., Wang L., Xue C.-Y., Zhang W.-D. (2011). Packaged silica microsphere-taper coupling system for robust thermal sensing application. Opt. Express.

[17] Tang T., Wu X., Liu L., Xu L. (2016). Packaged optofluidic microbubble resonators for optical sensing. Appl. Opt..

[18] Cosci A., Quercioli F., Farnesi D., Berneschi S., Giannetti A., Cosi F., Barucci A., Conti G.N., Righini G., Pelli S. (2015). Confocal reflectance microscopy for determination of microbubble resonator thickness. Opt. Express.

[19] Kamikawachi R.C., Abe I., Paterno A.S., Kalinowski H.J., Muller M., Pinto J.L., Fabris J.L. (2008). Determination of thermo-optic coefficient in liquids with fiber Bragg grating refractometer. Opt. Commun..

[20] Ward J.M., Dhasmana N., Chormaic S.N. (2014). Hollow core, whispering gallery resonator sensors. Eur. Phys. J. Spec. Top..

[21] Brenci M., Calzolai R., Cosi F., Nunzi G., Righini G.C., Pelli S. Microspherical resonators for biophotonic sensors. Proceedings of the Lightmetry and Light and Optics in Biomedicine 2004.

[22] Yang Y., Ward J., Chormaic S.N. (2014). Quasi-droplet microbubbles for high resolution sensing applications. Opt. Express.

[23] He L., Xiao Y.-F., Zhu J., Ozdemir S.K., Yang L. (2009). Oscillatory thermal dynamics in high-Q PDMS coated silica toroidal microresonators. Opt. Express.

[24] Ma Q., Rossmann T., Guo Z. Micro-temperature sensor based on optical whispering gallery mode of fiber taper-microsphere coupling system. Proceedings of the Photonic Fiber and Crystal Devices: Advances in Materials and Innovations in Device Applications III.

[25] White I.M., Oveys H., Fan X. (2006). Liquid-core optical ring-resonator sensors. Opt. Lett..

[26] Judy J., Maynes D., Webb B.W. (2002). Characterization of frictional pressure drop for liquid flows through microchannels. Int. J. Heat Mass Transf..

[27] Vollmer F., Arnold S. (2008). Whispering-gallery-mode biosensing: Label-free detection down to single molecules. Nat. Methods.

